# Aneurism by Anastomosis

**Published:** 1830-10-01

**Authors:** 


### ANEURISM BY ANASTOMOSIS.—BOTH CAROTIDS TIED.

DR. MUSSEY has published another case of this kind in the American Journal of Medical Sciences for February, 1830. The patient was a man 20 years of age, with a large purple pulsating tumour situated on the vertex of the head, having a sluggish ulcer at its apex, which occasionally bled. The tumour had existed since infancy, but increased greatly within the last three years. The left temporal artery and vein were much enlarged, and upwards of twenty arterial branches, each as large as a goose-quill, were seen actively pulsating on the scalp and running to the tumour. Dr. Mussey tied the left common carotid without success; on the 12th day he tied the right, and in four weeks the tumour diminished to a third of its original volume. After this it began again to increase, and astringent applications with pressure were had recourse to. On the 22d of November, six weeks from the second operation, Dr. Mussey removed the tumour altogether. This he did by making an incision round it through the soft parts, and then rapidly dissecting away the whole mass from the pericranium. Not more than an inch and a half of the scalp was divided at a time, and firm compression was made upon each lip of the incision while the vessels were secured by ligatures, more than forty of which were applied in making the circuit of the tumour. Notwithstanding these precautions nearly two quarts of blood were lost during the operation, which lasted more than an hour. The patient was faint, and continued so for some time. In eight weeks the wound was nearly healed, but some months elapsed before the cuticle became sufficiently firm. The patient returned to labour upon a farm in March or April, and has since continued most industrious and athletic. This adds another to the list of failures, from treating aneurism by anastomosis by tying the carotid arteries. We have argued the question again and again, and it now only remains to publish these cases from time to time, if surgeons will persist in performing these operations. We do not approve of excision of such large aneurismal tumours. The hæmorrhage in this case *was* dangerous, and *might* have been fatal. The ligature is preferable, and although there are few tumours so large as not to admit of it when ingeniously applied, yet even supposing there were such, a part of them might be tied and strangulated at a time. These are mere hints, for we have not space to enter more fully on the consideration of this very practical subject.

---

